# Beliefs and practices among primary care physicians during the first wave of the COVID-19 pandemic in Baden-Wuerttemberg (Germany): an observational study

**DOI:** 10.1186/s12875-021-01433-9

**Published:** 2021-05-06

**Authors:** Catharina Roth, Amanda Breckner, Sophia Moellinger, Simon Schwill, Frank Peters-Klimm, Joachim Szecsenyi, Sandra Stengel, Michel Wensing

**Affiliations:** Department of General Practice and Health Services Research, Heidelberg University Hospital, Marsilius Arcades, West Tower, Im Neuenheimer Feld 130, 69120 Heidelberg, Germany

**Keywords:** COVID-19, SARS-CoV-2, Primary Healthcare, Germany, Pandemic, Beliefs

## Abstract

**Background:**

During the first wave of the COVID-19 pandemic various ambulatory health care models (SARS-CoV-2 contact points: Subspecialised Primary Care Practices, Fever Clinics, and Special Places for Corona-Testing) were organised in a short period in Baden-Wuerttemberg, a region in Southern Germany. The aim of these SARS-CoV-2 contact points was to ensure medical treatment for patients with (suspected) and without SARS-CoV-2 infection. The present study aimed to assess the beliefs and practices of primary care physicians who either led a Subspecialised Primary Care Practice or a Primary Care Practice providing care as usual in Baden-Wuerttemberg during the first wave of the COVID-19 pandemic.

**Methods:**

This cross-sectional study was based on a paper-based questionnaire in primary care physicians during the first wave of the pandemic. Participants were identified via the web page of the Association of Statutory Health Insurance Physicians Baden-Wuerttemberg. The questionnaire was distributed in June and July 2020. It measured knowledge, practices, self-efficacy and fears towards SARS-CoV-2, using newly developed questions. Data was descriptively analysed.

**Results:**

One hundred fifty-five participants (92 leads of SARS-CoV-2 contact points/ 63 leads of primary care practices) completed the questionnaire. Out of 92 leads of SARS-CoV-2 contact points 74 stated to lead n Subspecialised Primary Care Practices. About half participants of both groups did not fear an own infection with the novel virus (between 50.8% and 62.2%), however about 75% feared financial loss. Knowledge was gained using various sources; main sources were the Association of Statutory Health Insurance Physicians (between 82.5% and 83.8%) and the German Society for Hygiene and Microbiology (RKI) (between 88.9% and 95.9%). Leads of Subspecialised Primary Care Practice felt more confident to perform anamnestic/diagnostic procedures (*p* < 0.001). The same was found for the confidence level regarding decision-making concerning the further treatment (*p* < 0.001). Several prevention measures to contain the spread of SARS-CoV-2 were adopted. Subspecialised Primary Care Practice had treated on average more patients with (suspected) COVID-19 (mean 408.12) than primary care practices (mean 83.8) (*p* < 0.001).

**Conclusion:**

The results of this study suggest that the Subspecialised Primary Care Practice that were implemented during the first wave of the SARS-CoV-2 pandemic contributed containment of the pandemic. Leads of Subspecialised Primary Care Practice indicated that physical separation of patients with potential SARS-CoV-2 infection was easier compared to those who continued working in their own practice. Additionally, leads of Subspecialised Primary Care Practice felt more confident in dealing with patients with SARS-CoV-2 infection.

**Trial registration:**

The study has been prospectively registered at the German Clinical Trial Register (DRKS00022224).

**Supplementary Information:**

The online version contains supplementary material available at 10.1186/s12875-021-01433-9.

## Background

The novel coronavirus (SARS-CoV-2) which causes the disease COVID-19 was first recognized in the Chinese province of Wuhan, Hubei in December 2019 [1]. The virus quickly spread into other provinces of China, to Thailand, Japan, South Korea, the USA, and Europe [2]. In Germany, the Bavarian Health and Food Safety Authority confirmed the first patient infected with SARS-CoV-2 infection on 27 January 2020 [3]. The effectiveness and resilience of health systems have an impact on the ability of a country to contain a pandemic [4, 5].

In Germany the primary healthcare sector consists of all ambulatory care services [6] that are provided by office-based, mainly single-handed, private general practitioners/primary care physicians, general internists or paediatricians. Almost half of the ambulatory care physicians are primary care physicians, the other half are other medical specialists (e.g. cardiologists, lung specialist), thus secondary care provider. Individuals can choose their primary care provider or their medical specialist freely [6]. Primary care physicians are remunerated based on the fee-for-service model or are paid a salary in rare cases (around 16 percent). According to that, physicians receive a fee for each service they provide e.g. office visits, test, procedures, or other healthcare services [7].

After the first case of COVID-19 was detected in Baden-Wuerttemberg, Germany on 25 February 2020, the Association of Statutory Health Insurance Physicians Baden-Wuerttemberg (Kassenaerztliche Vereinigung Baden-Wuerttemberg (KVBW)) asked all patients with suspected SARAS-CoV-2 infection to contact their primary care physician by telephone prior to visit a doctor [8]. Therefore, in Germany primary care physicians were responsible for a substantial part of the medical treatment of patients with (suspected) SARS-CoV-2 infection, comprising diagnostic testing, identification of those in need of hospital care, doing home visits, and supporting patients who manage the disease at home. At the same time, Germanys primary care sector had to ensure medical treatment for patients without a SARS-CoV-2 infection to prevent undersupply and were responsible to contain the spread of the virus within their practices and other health care facilities (e.g. nursing homes) [9]. Although Germany has a reasonably strong primary care sector and a well-organized public health system [10], many challenges need to be addressed for the preventive measures adopted to be effective during the pandemic. A qualitative study conducted in Australia, Israel, and England, for example, showed many issues during the 2009/A/H1N1 pandemic: challenges in patient’s consultation e.g. high flow of patients who thought they were infected needed to be treated, overall patient segregation was difficult to maintain, supply of personal protective equipment (PPE) was limited, communication of policies and guidelines, and an increased workload had an impact on ability to contain the pandemic [11]. The influenza pandemic plan of Baden-Wuerttemberg (BW) [9], a region with about 11 million inhabitants in South-West Germany, states that patients should be treated within the primary healthcare sector as long as possible. Additionally, patients who need to be hospitalized should be referred to primary care as soon as their state of health allows it [9].

In BW a variety of ambulatory health care models (SARS-CoV-2 contact points), besides the regular primary care practices, were established in a short period during the first wave of the SARS-CoV-2 pandemic as part of a crisis management: 204 Subspecialised Primary Care Practices, 51 Fever Clinics, and 16 Testing sites (Status: June 2020) by primary care physicians in cooperation with the public health sector and the Association of Statutory Health Insurance Physicians (German: Kassenaerztliche Vereinigung Baden-Wuerttemberg (KVBW)).

### SARS-CoV-2 contact points

Primary care practices should be, next to the public health organisations, the first contact point for patients if they are worried they may be infected with SARS-CoV-2 and show symptoms of the COVID-19 disease. In BW, three types SARS-CoV-2 contact points were established to support primary care physicians:

- Subspecialised Primary Care Practices, are primary care practices who offer appointments for patients with potential SARS-CoV-2 infection or other infectious diseases.
- Fever Clinics, which are usually located centrally or decentralised in local buildings (e.g. schools, sport halls) and are organised by primary care physicians and the KVBW.
- Special Points for Corona Testing, are available in some regions and are organised by the primary health care sector in collaboration with health authorities.

**Fig. 1:**
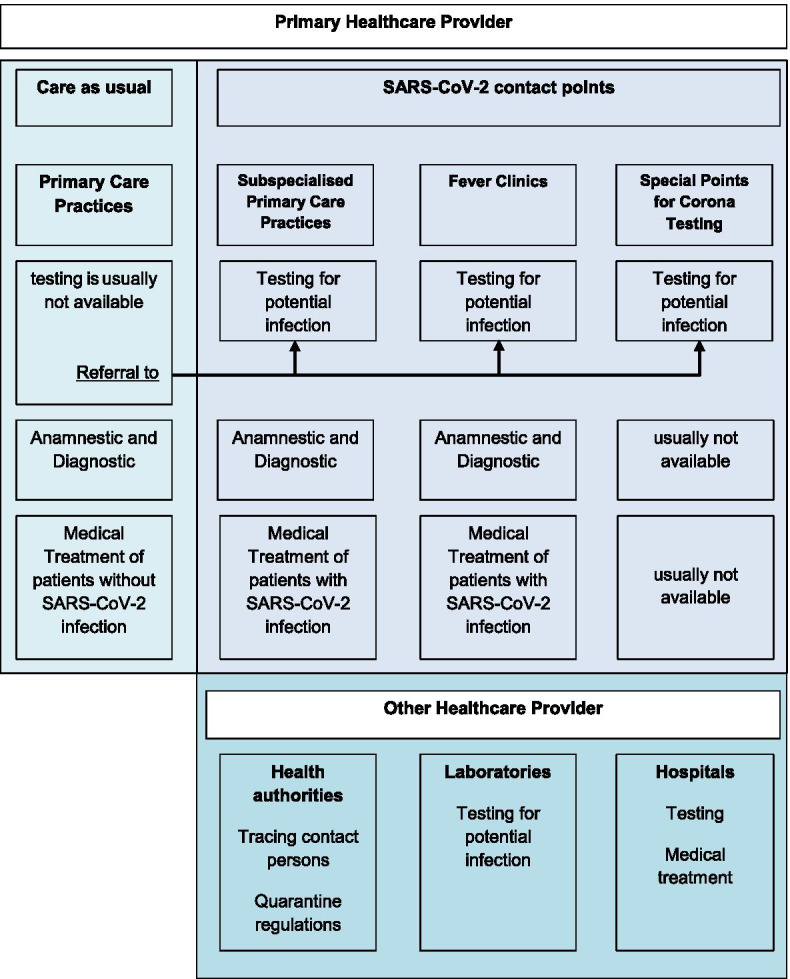
Organisation of the ambulatory sector in Baden-Wuerttemberg (Germany) during the COVID-19 pandemic (own figure)

The SARS-CoV-2 contact points allowed primary care physicians to refer patients with (suspects) SARS-CoV-2 infection in order to fulfil the requirement of patient separation (Fig. 1).

The newly implemented SARS-CoV-2 contact points are claimed to have an important role in the relatively moderate spread of the disease and the relatively low mortality by COVID-19 in Baden-Wuerttemberg, Germany during the first wave of the pandemic [12]. Little research has been done on the organisation of the newly implemented ambulatory healthcare models and the challenges primary care physicians had to face in the first months after the arrival of the virus in Germany.

The physicians` involvement in active participation in contact points was on a voluntary base, however incentivised by supply PPE in a time of extreme shortage in this regard. Therefore, the implementation of these ambulatory healthcare models was not systematically planned and the SARS-CoV-2 contact points were not equally spread over the country. The knowledge and beliefs of primary care physicians may influence the effective organisation of the different ambulatory health care models.

To get insights for further pandemic management, the present study aimed to assess beliefs such as the self-efficacy, practices, knowledge and fear among primary care physicians who decided to become a Subspecialised Primary Care Practices in comparison to primary care physicians who continued “usual” primary care. The other two SARS-CoV-2 contact points (*Fever Clinics and Special Points for Testing*) were excluded from the analysis due to the different settings and aims of those ambulatory models.

## Methods

This cross-sectional study was based on a paper-based survey in primary care physicians who worked in Subspecialised Primary Care Practices in BW, Germany as well as primary care practices who continued to provide care as usual during the first wave of the SARS-CoV-2 pandemic.

### Ethical considerations

The study was registered at the German Clinical Trial Register prior to the start of the study, registration number: DRKS00022224. The ethical committee of the medical faculty of the Heidelberg University approved the study (S-418/2020). Informed consent to participate was obtained from all participants when they posted the completed questionnaire to the research team. The research conducted in this study was performed in accordance with the Declaration of Helsinki.

### Recruitment and study sample

All 271 SARS-CoV-2 contact points in BW were identified via the web page of the Association of Statutory Health Insurance Physicians Baden-Wuerttemberg (Kassenaerztliche Vereinigung Baden-Wuerttemberg (KVBW)) and invited to participate in this survey in June 2020. Included were all primary care physicians who led one of the SARS-CoV-2 contact points. The aim was a full census of primary care physicians leading a SARS-CoV-2 contact point in BW during the first wave of the SARS-CoV-2 pandemic (March—June 2020). No inclusion criteria were set regarding the time point of the establishment of the SARS-CoV-2 contact points. They only had to have operated during the first wave of the pandemic. Furthermore, a random sample of 400 other primary care practices, also identifies via the web page of the KVBW, was invited to participate in the study. The invitation was addressed to the practice owner of the primary care practice. In case of an group practices, physicians were given the option to complete the questionnaire together or to decided who would be best to complete the questionnaire. Primary physicians of those were excluded from the analysis, if they indicated that they also worked for a SARS-CoV-2 contact point.

### Questionnaire

The questionnaire (Additional file 1, German) was developed at the Department of General Practice and Health Services Research at the University Hospital Heidelberg and based on eight telephone interviews with primary care physicians to identify relevant topics. In total six relevant topics were identified. The first part of the questionnaire focused on demographic and practice characteristics such as age, gender, professional qualification, type of SARS-CoV-2 contact point, inhabitants, catchment area, location, and month of implementation (*in categories*). The second part of the questionnaire covered the structure and organisation of the SARS-Cov-2 contact points/ primary care practices including questions such as number of staff (in *total numbers*), opening hours, changes to opening hours, remuneration, spatial conditions, diagnostic possibilities, treatment offers, the financing of the SARS-CoV-2 contact, and type of support which enable the implementation (*in categories*). The third part comprised patients contacts and treatment capacities (*in total numbers*). Part four covered questions regarding the nature of the medical documentation and medical history used, the main content of medical documentation used, and the satisfaction with these documents (*answering options yes, partly, no, I don’t know*). This part also included questions concerning collaboration/cooperation with other healthcare facilities (*in categories*) and satisfaction with it (*answering options yes, partly, no, I don’t know*).

The main part of the questionnaire, which is reported in this paper, covered questions regarding fear for an infection, self-efficacy and practice, sources (*in categories*) and level of knowledge (*answering options yes, partly, no, I don’t know*). The last part focused on PPE and disinfection methods (*in categories*), other prevention measures, the utilisation of their SARS-CoV-2 contact point/ primary care practices and how healthcare during the second wave of the SARS-CoV-2 pandemic should be managed (*answering options yes, partly, no, I don’t know*) in BW, Germany. The last question was an open-end question which gave the participants the opportunity to share their personal thoughts regarding the pandemic.

### Data collection

Each primary care physician leading a SARS-CoV-2 contact point as well as a random sample of other primary care physicians who owned the practice were invited to participate in the paper-based survey. They received an information leaflet, the paper-based questionnaire, a reply envelope, and a letter from the KVBW with the request to participate. Data collection was conducted between 15 June and 20 July 2020. A reminder was sent to all potential participants two weeks after the initial invitation to the survey.

### Data analysis

Data was analysed using the statistic software IBM SPSS Version 25.0. Mean and standard deviations for continuous variables and frequencies and percentages for categorial variables were calculated. Chi-Square tests were used to examine if differences in SARS-CoV-2 contact points and primary care practices were significant. For continuous variables a student’s t-test was conducted. *P* < 0.05 was considered significant in all analysis.

The aim of this study was to describe and compare Subspecialised Primary Care Practices and Primary Care Practices which provided care as usual during the first wave of the SARS-CoV-2 pandemic.

## Results

Out of 271 SARS-CoV-2 contact points 92 participated (16 Fever Clinics, 74 Corona-Subspecialised Primary Care Practices, and 2 Special Places for Corona-Testing) (responds rate 33.9%). Of the 400 invited primary care physicians 79 participated (responds rate 19.7%), 16 of those were excluded from the analysis since they indicated that they also worked for a SARS-CoV-2 contact point.

Table 1 shows that leads of Subspecialised Primary Care Practices were predominantly male (66.2%) and between 51 and 60 years old (40.5%), with a qualification of primary care medicine compared to other medical specialists. Almost 50% of the primary care physicians, were female and also predominantly between 51 and 60 years old. Most Subspecialised Primary Care Practices were located in places with a number of inhabitants between 5000 and 20,000, covering a catchment area between 15 and 30 kms. Primary Care Practices were mainly located in city centres with inhabitants between 5000 and 200,000. Most Subspecialised Primary Care Practices opened in March 2020, the first month of the SARS-CoV-2 pandemic in Germany. There was a difference between male and female physicians between the two groups (x^2^ = 4.482, *p* = 0.034). Other sociodemographic factors such as age or professional qualification were not significant (Table 1).

**Table 1:** Description of the study population

Characteristics	Corona-Subspecialised Primary Care Practices (*n* = 74)	Primary Care Practices (*n* = 63)
**Age group, n (%)**		
Under 30 years	0	0
Between 30 and 40 years	11 (14.9)	7 (11.1)
Between 41 and 50	23 (31.1)	16 (25.4)
Between 51 and 60	30 (40.5)	26 (41.3)
Above 60 years	9 (12.2)	14 (22.2)
No answers	1 (1.4)	0
**Gender, n (%)**		
Male	49 (66.2)	31 (49.1)
**Professional qualification, n (%)**^a^		
Primary care physicians	74 (100.0)	60 (95.24)
Other medical specialists	16 (21.6)	11 (14.5)
No answer	0	3 (4.8)
**Number of inhabitants, n (%)**		
Less than 5.000 inhabitants	11 (14.9)	3 (4.8)
Between 5.000 and 20.000 inhabitants	35 (47.3)	24 (38.1)
Between 20.000 and 100.000 inhabitants	15 (20.3)	23 (36.5)
Over 100.000 inhabitants	13 (17.6)	13 (20.6)
**Catchment area, n (%)**		
Less than 15 km	10 (13.5)	n/a
Between 15 and 30 km	65 (75.7)	
Between 30 and 50 km	6 (8.1)	
More than 50 km	2 (2.7)	
**Location, n (%)**		
City centre	30 (40.5)	36 (57.1)
Urbanized (20 km)	27 (36.5)	17 (27.0)
Rural area (City > 20 km)	17 (23.0)	8 (12.7)
No answer	0	2 (3.2)
**Implementation of the SARS-CoV-2**		
February 2020	4 (5.4)	n/a
March 2020	35 (47.3)	
April 2020	25 (33.8)	
May 2020	4 (5.4)	
June 2020	1 (1.4)	
No answer	5 (6.8)	

^a^ Multiple answer were possible

### Fears related to SARS-CoV-2

Primary care physicians who worked in Primary Care Practices seemed to feel slightly more anxious towards an own infection compared to their peers who worked in Subspecialised Primary Care Practices (25.4% compared to 11.9%). Primary care physicians, stated to be worried for various reason like transmitting the virus to the private environment (42.9%) or the professional environment (46.0%). In comparison, primary care physicians who led a Subspecialised Primary Care Practices tended to feel less anxious to fall sick themselves (62.2%) or to spread the virus within their private environment (37.8%) or their professional environment (45.9%). The vast majority of participants of the regular Primary Care Practices and Subspecialised Primary Care Practices (between 75.7 and 84.1%) feared financial loss during the pandemic because of a possible closure of practices and therefore loss of income. However, none of the differences between the two groups were significant (Table 2).

**Table 2:** Fear for a potential infection with SARS-CoV-2 or financial loss due to practice closure

	Corona-Subspecialised Primary Care Practices **(*n* = 74)**	Primary Care Practices ***n = 63***	***p-Value***
I fear for an infection with SARS-CoV-2 because I may develop COVID-19 **myself**			
Yes	11 (14.9)	16 (25.4)	0.276
Partly	17 (23.0)	14 (22.2)	
No	46 (62.2)	32 (50.8)	
I don’t know/ no answer	0	1 (1.6)	
I fear for an infection with SARS-CoV-2 because I may **spread the virus** within my private environment			
Yes	20 (27.0)	27 (42.9)	0.108
Partly	25 (33.8)	12 (19.0)	
No	28 (37.8)	24 (38.1)	
I don’t know/ no answer	1 (1.4)	0	
I fear for an infection with SARS-CoV-2 because I may **spread the virus** within my professional environment			
Yes	22 (29.7)	29 (46.0)	0.194
Partly	17 (23.0)	13 (20.6)	
No	34 (45.9)	21 (33.3)	
I don’t know/ no answer	1 (1.4)	0	
I fear for a financial loss due a temporary practice closure due to an infection with SARS-CoV-2			
Yes	56 (75.7)	53 (84.1)	0.153
No	18 (24.3)	9 (14.3)	
I don’t know/ no answer	0	1 (1.6)	

### Source and level of knowledge

Participants were also asked about their sources and their level of knowledge during the first wave of SARS-CoV-2 pandemic (Table 3). At the time of the survey the majority of participants (between 88.9 and 91.9%) indicated that their level of knowledge was up to date. At the beginning of pandemic for 77% of leads of Subspecialised Primary Care Practices and 65.1% of the primary care physicians stated that meeting their needs for knowledge regarding SARS-CoV-2 was difficult. This changed during the ongoing pandemic. At present only 43.2% of leads of Subspecialised Primary Care Practices and 34.9% stated that addressing their knowledge regarding SARS-CoV-2 was difficult. Information was used from various sources; main sources were the Association of Statutory Health Insurance Physicians, the German Society for Hygiene and Microbiology (RKI), the German federal Government Agency, and research institute responsible for disease control and prevention. The novel source of knowledge “podcasts” was used by around 40% of all participants. None of the differences between the groups were significant (Table 3).

**Table 3:** Knowledge and sources of knowledge related to the SARS-CoV-2 pandemic

	Corona-Subspecialised Primary Care Practices **(*n* = 74)**	Primary Care Practices ***n = 63***	***p-value***
My level of knowledge regarding SARS-CoV-2 is up to date ^b^	68 (91.9)	56 (88.9)	0.831
To address my lack of knowledge regarding SARS-CoV-2 was difficult at the beginning of the first wave of the pandemic ^b^	57 (77.0)	41 (65.1)	0.332
To address my lack of knowledge regarding SARS-CoV-2 is difficult at present ^b^	32 (43.2)	22 (34.9)	0.607
**Sources of knowledge are**^a^			
Association of Statutory Health Insurance Physicians	62 (83.8)	52 (82.5)	n/a
Robert Koch Institute (RKI) *(German Society for Hygiene and Microbiology)*	71 (95.9)	56 (88.9)	
Health Authorities	30 (40.5)	25 (39.7)	
DEGAM	39 (52.7)	29 (46.0)	
Hausaerzte Verband *(German Federation of General Practitioners)*	25 (33.8)	28 (44.4)	
Colleagues	34 (45.9)	26 (41.3)	
Medical Chamber	12 (16.2)	14 (22.2)	
Deutsches Aerzteblatt *(Official journal of the German Medical Association.)*	37 (50.0)	40 (63.5)	
Deximed *(German Medical Encyclopaedia)*	8 (10.8)	6 (9.5)	
Podcast designated virologist (Berlin)	31 (41.9)	23 (36.5)	
Podcast designated virologist (Halle)	10 (13.5)	5 (7.9)	
Podcast chair of DEGAM	6 (8.1)	5 (7.9)	
Other Podcast	n/a	6 (9.5)	
Journals	44 (59.5)	38 (60.3)	
Other	20 (27.0)	11 (17.5)	

^a^ Multiple answer were possible

^b^ Answering options were yes, partly, no, I don’t know/ no answer; for better readability only ‘yes’ is reported

### Self-efficacy regarding anamnestic and diagnostic procedures related to SARS-CoV-2

Another part of the survey was the self-efficacy and practice of participants regarding anamnestic and diagnostic procedures related to SARS-CoV-2 and the decision-making process regarding the further procedure. The results show that in both settings the confidence to treat patients with (suspected) SARS-CoV-2 infection increased over time. There was a difference in the confidence level between the two groups in performing anamnestic and diagnostic procedures for patients with COVID-19 at the beginning of the pandemic (x^2^ = 19.374, *p* = 0.001), after four weeks (x^2^ = 27.571, *p* < 0.001), and present (x^2^ = 11.288, *p* = 0.024). This result suggests that primary care physicians who led a Subspecialised Primary Care Practice felt significantly more confident. The same is true for the confidence level regarding the decision-making process regarding further procedure. At the beginning of the pandemic and after four weeks there was a difference between the both groups (x^2^ = 13.074, *p* = 0.011, x^2^ = 20.298, *p* < 0.001, respectively). There was no significant difference regarding deciding how the further procedure of patients with COVID-19 should look like when the participants completed the questionnaire (June/July 2020) (Table 4).

**Table 4:** Self-efficacy regarding anamnestic and diagnostic procedures related to SARS-CoV-2

	Corona-Subspecialised Primary Care Practices **(*n* = 74)**	Primary Care Practices ***n = 63***	***p-value***
At the beginning of my work as Subspecialised Primary Care Practice/ of the pandemic (Primary care practices) I felt confident in performing anamnestic and diagnostic procedures for patients with COVID-19 ^a^	44 (59.5)	24 (38.1)	0.001
After 4 weeks of working at the SARS-CoV-2 contact point/ in the Easter period (Primary care Practices) I felt confident in performing anamnestic and diagnostic procedures for patients with COVID-19 ^a^	66 (89.2)	35 (55.6)	< 0.001
At present I feel confident in performing anamnestic and diagnostic procedures for patients with COVID-19 ^a, b^	66 (89.2)	48 (76.2)	0.024
At the beginning of my work at the SARS-CoV-2 contact point/ of the pandemic (Primary care practice) I feel confident to decide how the further procedure for patients with COVID-19 should look like ^a^	40 (54.1)	26 (41.3)	0.011
After 4 weeks of working at the SARS-CoV-2 contact point/ in the Easter period (Primary care practice) I feel confident to decide how the further procedure for patients with COVID-19 should look like ^a^	66 (89.2)	40 (63.5)	< 0.001
At present I feel confident to decide how the further procedure for patients with COVID-19 should look like ^a, b^	67 (90.5)	56 (88.9)	0.594

^a^ Answering options were yes, partly, no, I don’t know/ no answer; for better readability only ‘yes’ is reported

^b^ Data was collected between 15 June and 20 July 2020

### Adoption of prevention measures and hygiene regulations

Different prevention measures to contain the spread of SARS-CoV-2 were adopted in both settings. The majority of leads of Subspecialised Primary Care Practice points and almost all primary care physicians reported that the implementation of hygiene regulations (masks, social distancing, disinfection) was possible (95.9 and 87.3%, respectively). However, the difference was not significant (x^2^ = 5.326, *p* = 0.070). The vast of majority of all participants stated that physical and temporal separation of patients’ groups was done. However, there was a significant difference regarding physical separation, suggesting that leads of Subspecialised Primary Care Practice points tended to feel like introducing physical separation was implemented easier (x^2^ = 10.925, *p* = 0.012). Physical separation was ensured by using different waiting or treatment rooms, waiting times in cars or in front of the facility. Temporal separation was ensured by appointment allocation (Table 5).

**Table 5:** Prevention measures to contain the spread of SARS-CoV-2

	Corona-Subspecialised Primary Care Practices **(*n* = 74)**	Primary Care Practices ***n = 63***	***p-value***
The implementation of hygiene regulations regarding the contact with patients with potential SARS-CoV-2 infection in our facility was possible ^b^	71 (95.9)	55 (87.3)	0.070
Medical face masks were available sufficient number ^b^	73 (98.6)	56 (88.9)	0.094
The physical separation of patients with potential SARS-CoV-2 infection was possible ^b^	66 (89.2)	46 (73.0)	0.012
The temporal separation of patients with potential SARS-CoV-2 infection was possible ^b^	62 (83.8)	47 (74.6)	0.717
**Physical separation was ensured by**^a^			
Different waiting rooms	18 (24.3)	19 (30.1)	n/a
Waiting time in the car in front of the facility	41 (55.4)	22 (34.9)	
Waiting time in front of the facility	53 (71.6)	39 (61.9)	
Different treatment rooms	55 (74.3)	19 (30.1)	
Waymarks	18 (24.3)	6 (9.5)	
Other	23 (31.1)	44 (69.8)	
No answer	3 (4.1)	1 (1.6)	
**Temporal separation ensured by**^a^			
Appointment allocation	66 (89.2)	n/a	n/a
Overall less patients	n/a	26 (41.3)	
Special days for patients with SARS-CoV-2 infection	n/a	1 (1.6)	
Scheduled time for consultations for patients with SARS-CoV-2 infection	n/a	46 (73.0)	
Other	13 (17.6)	12 (19.05)	
No answer	5 (6.8)	7 (11.1)	

^a^ Multiple answer were possible

^b^ Answering options were yes, partly, no, I don’t know/ no answer; for better readability only ‘yes’ is reported

### Treatment capacity and patient contacts

Subspecialised Primary Care Practice treated on average 2 more patients with (suspected) COVID-19 (mean 7.70 (0–50)) than primary care practices (mean 5.70 (0–120)). However, the difference was not significant. In total Subspecialised Primary Care Practice treated on average 408.12 (0–3846) patients since they opened, whereas on average 83.8 patients were treated by primary care practices (F (151.447 – 501.469) = 19.614, *p* < 0.001). The maximum treatment capacity of Subspecialised Primary Care Practice can be increase immediately on average by 25.32 patients, if personal resources are increased by 25.70 patients, or if other actions are adapted by 43.41 patients. Noticeable is the range of variation in treating patients between individual participants during the first wave of the pandemic in both groups (Table 6).

**Table 6:** Treatment capacity per Corona-Subspecialised Primary Care Practice and Primary Care Practice during the first wave of the SARS-CoV-2 pandemic in Baden-Wuerttemberg, Germany

	Corona-Subspecialised Primary Care Practices ***N = 64***	Primary Care Practices ***N = 44***	***p-value***
**Treatment of patients with or with potential SARS-CoV-2 infection**			
Average per day ^a^	7.70 (0–50)	5.7 (0–120)	0.665
Total since opening ^a^	408.12 (0–3846)	83.8 (2–500)	< 0.001
**Maximum treatment capacity for patients with or with potential SARS-CoV-2 infection per day**			
	***N = 74***		
Immediately ^a^	25.32 (0–140)	n/a	
If personal resources are increased ^a^	25.70 (0–200)		
If other actions are adapted ^a^	43.41 (0–500)		

^a^ Reported are mean, minimum, and maximum. Responds option average per day estimated or calculated, as well as overall since opening were summarized using either the calculated values of the practice software (if reported) or the estimated values

## Discussion

The main goal of this study was to describe beliefs such as knowledge, practice, self-efficacy and fears among primary care physicians leading a Subspecialised Primary Care Practice compared to primary care physicians who “continued usual primary care practice” during the first wave of COVID-19 pandemic in Baden-Wuerttemberg, Germany. A quarter of all participating primary care physicians did not fear an infection with SARS-CoV-2 but were afraid of transmitting it to members of their families or colleagues. The majority of all participants was afraid of financial loss due to lost revenues. Acquiring knowledge about the new coronavirus disease was challenging but this changed during the pandemic and extending knowledge. Sources of knowledge varied but the Association of Statutory Health Insurance Physicians, the German Society for Hygiene and Microbiology, and medical journals represented the most important sources of information. The majority of primary care physicians of both groups felt confident in anamnestic and diagnostic procedures. Primary care physicians who led a Subspecialised Primary Care Practice were more confident compared to their peers. Hygiene regulations were implemented in all healthcare facilities. Physical separation was ensured mainly by different treatment rooms and waiting in front of the facility by both groups but the results of this study showed that leads of Subspecialised Primary Care Practice tended to introduce physical separation easier. Temporal separation was ensured via appointment allocation, overall fewer patients in Primary Care Practices or special consultation hours for patients with suspected infection. Subspecialised Primary Care Practice treated on average significant more patients with (suspected) COVID-19 compared to Primary Care Practices.

The results of this study showed that most participants did not fear an infection with the novel virus but expressed concerns regarding the possibility to spread it within their professional and private environment. It is unclear why some decided to lead a Subspecialised Primary Care Practice and some continued usual primary care practice. Interestingly, the majority of physicians who led a Subspecialised Primary Care Practice identified as male and were between 51 and 60 years old and therefore more at risk for a severe infection [13]. In Germany in general more than half of the primary care physicians are male (54.1%) [14] and are on average 55.4 years old [15]. We can only speculate about reasons of the higher participation in Subspecialised Primary Care Practice among older male physicians: Perhaps is has to do with gender differences in health oriented behavior in general. Further studies would be preferable to answer that delicate question.

The majority of both groups feared financial lose. This could be explained by the concept of fee-for-service, particularly physicians who continued to work in their own practices had overall less patients which may had an impact on the fear of financial lose. These results agree with those by Huston et al. [5]. In Australia and New Zealand for example were remuneration is mainly based on fee-for-service a rapid decrease in patient visits has led to severe financial losses [5]. The leads of Subspecialised Primary Care Practice in this study also feared financial loss. Because of the dynamic development of the Subspecialises Primary Care Practices and also the clarifying of the remuneration in the course it is unclear if results remain the same in a second survey.

In order to separate patients with potential infections they introduced special consultation hours (appointment allocation) and waiting times e.g. in front of the practice. Offering special consultation hours may had impact on their workload that was not adequately remunerated. In addition, waiting hours in front of the practice may had an impact on the decision of patients to consult their physicians at all. The specific concerns of primary care physicians should be taken into consideration since primary healthcare systems and public health systems rely on the mental and physical health of primary care physicians and their economic efficiency [16].

The majority used the Association of Statutory Health Insurance Physicians and the German Society for Hygiene and Microbiology as source of knowledge which provide an easy and accessible way of getting valid and processed information regarding a growing and changing knowledge base. Interestingly, almost half of the participants (between 36 and 42%) stated to listen to a podcast even though the majority of participants were in the age groups between 41 and 60 years. Podcasts or broadcasts are primarily used by the younger generation (between 14 and 29 years) [17]. Another reason might be the trust in a designated virologist subspecialised in Coronaviruses who chose to record an own podcast to inform the broader community about SARS-CoV-2. Interesting is that only half used information provided by professional societies.

The vast majority of primary care physicians in this study used official information provided by the Association of Statutory Health Insurance Physicians and the RKI. This may can be interpreted, as trust in official information channels by the participants. Nevertheless, various other sources of information were used additionally. This poses the questions if the participants probably felt like information of official channels may be not enough or if those sources may not suitable for the primary care sector. In addition, gathering knowledge was difficult for more than half of the participants at the beginning of the pandemic and was still evaluated as challenging by a third during the pandemic. This may made it difficult to be always up-to-date. The diversity of potential information resources and the need of being up-to-date probably had an impact on the perceived workload of primary care providers. Moreover, primary care physicians are in direct contact with patients with (suspected) SARS-CoV-2 infection and are therefore at high risk for nosocomial infections [18]. During a health crisis such as a global pandemic, primary healthcare providers need to be equipped with up-to-date-knowledge in order to practice safely and efficiently. Further, research needs to be conducted in order to evaluate which source of information is the most feasible for primary care providers and which is the preferred way of reviving information.

Self-efficacy regarding anamnestic and diagnostic procedures related to SARS-CoV-2 of primary care physicians increased over time during the first wave of the pandemic. This can be explained by the experience they gained during the first month of the pandemic. Physicians had to acquire knowledge on the novel virus first under the permission of the dynamic situation of the pandemic. For a second wave of SARS-CoV-2 pandemic primary care physicians may be better prepared since scientific as well practical knowledge improved during the first months of the pandemic. The findings of this study indicated that although self-efficacy increased over time in both groups, leads of Subspecialised Primary Care Practice felt more confident. This may be important in order to decide which structure of medical care and treatment is the most favourable during the second wave of the SARS-CoV-2 pandemic or other pandemics.

The results of our study showed that key challenges which were difficult to tackle during the 2009/A/H1N1 pandemic in Australia, Israel, and England such as segregation of patients with (suspected) infection [11] were easier implemented during this pandemic in Baden-Wuerttemberg, Germany in both settings. A study conducted in Germany during the influenza pandemic 2009/10 [16], for example, showed that changing their practice management in order to separate patients physically and in time was implemented by 74% and 38% of the participants, respectively. However, participants in this study [16] were part of the surveillance network of the German Society for Hygiene and Microbiology which may implied a selection bias. Nevertheless, physical segregation of patients was easier for primary care physicians who led a Subspecialised Primary Care Practice. Further research is needed to evaluated why this was the case.

First evaluations on accounting data have shown that seven out of eight COVID-19 patients (about 85%) were treated in an ambulatory setting [19, 20]. In our study, the average treatment capacity of patients with (suspected) SARS-CoV-2 infection varies between the different two groups. On average more patients were treated at Subspecialised Primary Care Practice.

Although the results of our study cannot be generalized, they may give a first impression on the workload primary care physicians had to manage in Baden-Wuerttemberg, Germany. As requested and expected more patients with (suspected) COVID-19 were treated in Subspecialised Primary Care Practice than in Primary Care Practices. The implementation of Subspecialised Primary Care Practice as well as the prevention measures in both settings may have contained the spread of the virus.

### Limitations

Although this study gives first impressions and important information on challenges primary care physicians had to face during the first wave of the SARS-CoV-2 pandemic, limitations on the study have to be considered. The analysis is based on self-disclosures, so biases cannot be excluded. The response rate is similar to other studies in German physicians, but it may imply selection bias. Furthermore, it is unclear whether the results can be generalized to Germany as the study concerns one region.

## Conclusion

The results of this study showed that the Subspecialised Primary Care Practice implemented during the first wave of the SARS-CoV-2 pandemic in Baden-Wuerttemberg, Germany were a strategy to face the pandemic. Particularly, since leads of Subspecialised Primary Care Practice indicated that implementing physical separation of patients with potential SARS-CoV-2 infection was easier compared to those who continued working in their own practice. In addition, primary care physicians who lead a Subspecialised Primary Care Practice felt in general more confident in dealing with patients with COVID-19 infection. Although, primary care physicians rose to the challenges of the SARS-CoV-2 pandemic, addressing their fears is essential. Interventions such as adequate payment for COVID-19 care or loss of revenue due to the pandemic combined with measures that support the implementation of prevention measure not only in Subspecialised Primary Care Practice but also Primary Care Practices should be introduced to support the primary health care sector.

Future research should focus on strategies how primary care physicians can be better prepared for a pandemic and how to address mental, physical and financial challenges. This could be for example the expansion of adequately financially compensated points of care, support by tools for managing testing, communication with and care of patients, setting up paths of cooperation between primary care, hospital care, rescue service and health authorities. Primary care physicians will be stressed by the challenges of a prolonged response to the SARS-CoV-2 pandemic. Strengthening primary healthcare, particularly primary care physicians, is therefore of great importance.

### Lessons learned and Implications for future practice

The results of this study indicated that primary care physicians who continued with “care as usual” did fear an infection with SARS-CoV-2 compared to physicians who lead a Subspecialised Primary Care Practice. Although the difference was not significant, for the second wave of the pandemic or other pandemics a list with primary care providers who are willing to open a Subspecialised Primary Care Practice and feel more confident in doing so should be developed. Additionally, Primary Care Practices that can implement prevention measures or already implemented them should be systematic listed and functioning as pandemic response practices. An information system with the latest state of scientific knowledge regarding the pandemic should be develop based on the preferences of primary care providers in order to support them to practice safely and to be up-to-date without an increased workload.

## Supplementary Information

**Additional file 1**

## Data Availability

The datasets generated and/or analysed during the current study are not publicly available due to European Data Protection Law but are available from the corresponding author on reasonable request.
