# Supplementary material for: Beliefs and practices among primary care physicians during the first wave of the COVID-19 pandemic in Baden-Wuerttemberg (Germany): an observational study
Source: BMC Fam Pract. 2021 May 6;22:86. doi: 10.1186/s12875-021-01433-9 (PMC8099434; doi:10.1186/s12875-021-01433-9)
Supplement: Supplementary file 1 — Additional file 1 [file 12875_2021_1433_MOESM1_ESM.pdf]

Additional File 1

Beliefs and practices among primary care physicians during the first wave of the COVID-19 pandemic in Baden-Wuerttemberg (Germany): an observational study

Catharina Roth[1], Amanda Breckner[1], Sophia Moellinger[1], Simon Schwill[1], Frank Peters-Klimm[1], Joachim Szecsenyi[1], Sandra Stengel[1], Michel Wensing[1]

1 Heidelberg University Hospital, Department of General Practice and Health Services Research, Marsilius Arcades, West Tower, Im Neuenheimer Feld 130, 69120 Heidelberg, Germany

Catharina Roth GKP. B.Sc. MPH*<br><br>Corresponding author	Department of General Practice and Health Services Research<br>Heidelberg University Hospital, Marsilius Arcades, West Tower, Im Neuenheimer Feld 130.3, D-69120 Heidelberg<br>Email: catharina.roth@med.uni-heidelberg.de
Amanda Breckner M.A.*	Department of General Practice and Health Services Research<br>Heidelberg University Hospital, Marsilius Arcades, West Tower, Im Neuenheimer Feld 130.3, D-69120 Heidelberg<br>Telefonnummer: +49 6221/ 56 34646<br>E-Mail: amanda.breckner@med.uni-heidelberg.de
Sophia Moellinger B.Sc.	Department of General Practice and Health Services Research<br>Heidelberg University Hospital, Marsilius Arcades, West Tower, Im Neuenheimer Feld 130.3, D-69120 Heidelberg<br>Email: Sophia.Moellinger@med.uni-heidelberg.de
Dr. med. Simon Schwill	Department of General Practice and Health Services Research<br>Heidelberg University Hospital, Marsilius Arcades, West Tower, Im Neuenheimer Feld 130.3, D-69120 Heidelberg<br>Email: simon.schwill@med.uni-heidelberg.de
Prof. Dr. med. Frank Peters-Klimm	Department of General Practice and Health Services Research<br>Heidelberg University Hospital, Marsilius Arcades, West Tower, Im Neuenheimer Feld 130.3, D-69120 Heidelberg<br>Email: frank.peters-klimm@med.uni-heidelberg.de
Prof. Dr. med. Joachim Szecsenyi. Dipl. Soz.	Department of General Practice and Health Services Research<br>Heidelberg University Hospital, Marsilius Arcades, West Tower, Im Neuenheimer Feld 130.3, D-69120 Heidelberg<br>Email: joachim.szecsenyi@med.uni-heidelberg.de
Dr. med. Sandra Stengel	Department of General Practice and Health Services Research<br>Heidelberg University Hospital, Marsilius Arcades, West Tower, Im Neuenheimer Feld 130.3, D-69120 Heidelberg<br>Email: sandra.stengel@med.uni-heidelberg.de
Prof. Dr. Michel Wensing. M.Sc.	Department of General Practice and Health Services Research<br>Heidelberg University Hospital, Marsilius Arcades, West Tower, Im Neuenheimer Feld 130.3, D-69120 Heidelberg<br>Email: michel.wensing@med.uni-heidelberg.de

* These authors contributed equally

Questionnaire: Primary Care Practice

Part 1: Demographic and Practice Characteristics

**Bitte geben Sie das heutige Datum an.**<br>*z.B. 12.03.2020*	
**Soziodemographie**	
**Welcher Altersgruppe gehören Sie an?**	☐ < 30 Jahre<br>☐ 30 bis 40 Jahre<br>☐ 41 bis 50 Jahre<br>☐ 51 bis 60 Jahre<br>☐ 61 bis 70 Jahre<br>☐ > 70 Jahre
**Welchem Geschlecht fühlen Sie sich zugehörig?**	☐ Weiblich<br>☐ Männlich<br>☐ Divers<br>☐ Andere: ______________________________

**Struktur/Organisation**	
**Wie viele Einwohner hat der Ort, in dem sich Ihre Einrichtung befindet?**	☐ < 5000 Einwohner*innen<br>☐ 5000 - < 20.000 Einwohner*innen<br>☐ 20.000 - < 100.000 Einwohner*innen<br>☐ 100.000 und mehr Einwohner*innen<br>☐ Kann ich nicht beurteilen
**Wo liegt Ihre Hausarztpraxis?**	☐ Stadtzentrum<br>☐ Stadt-Umkreis (20km)<br>☐ Ländliches Gebiet (Stadt > 20km)
**Welche berufliche Qualifikation haben Sie?**<br>*(Mehrfachnennung möglich)*	☐ Fachärztin/-arzt für Allgemeinmedizin<br>☐ Fachärztin/-arzt für Innere Medizin<br>☐ Sonstiges:
**Ich bin auch in einer Corona-Anlaufstelle (Abstrichstelle, Corona-Schwerpunktpraxis, zentrale Fieberambulanz) tätig oder tätig gewesen.**	☐ Ja<br><br>☐ Nein
**Falls ja – bitte füllen Sie den Fragebogen nicht weiter aus und informieren Sie uns per Mail (Kontaktdaten siehe letzte Seite), dass Sie nicht an der Studie teilnehmen können. Hintergrund ist, dass es zu Verzerrungen kommen könnte, wenn Sie parallel zu Ihrer hausärztlichen Tätigkeit in einer Corona-Anlaufstelle tätig sind.**<br><br>**Vielen Dank!**	

© Universitätsklinikum Heidelberg 2020

Part 2: Fear for a potential infection with SARS-CoV-2 or financial loss due to practice closure

Einstellung/ Haltung	
**Ich habe Angst, mich bei meiner Tätigkeit mit SARS-CoV-2 anzustecken, weil ich selbst erkranken könnte.**	☐ Trifft ganz zu<br>☐ Trifft weitgehend zu<br>☐ Trifft teils zu, teils nicht<br>☐ Trifft weitgehend nicht zu<br>☐ Trifft überhaupt nicht zu<br>☐ Kann ich nicht beurteilen
**Ich habe Angst, mich bei meiner Tätigkeit mit SARS-CoV-2 anzustecken, weil ich andere im privaten Umfeld anstecken könnte.**	☐ Trifft ganz zu<br>☐ Trifft weitgehend zu<br>☐ Trifft teils zu, teils nicht<br>☐ Trifft weitgehend nicht zu<br>☐ Trifft überhaupt nicht zu<br>☐ Kann ich nicht beurteilen
**Ich habe Angst, mich bei meiner Tätigkeit mit SARS-CoV-2 anzustecken, weil ich andere im beruflichen Umfeld (Patient*innen, Mitarbeitende) anstecken könnte.**	☐ Trifft ganz zu<br>☐ Trifft weitgehend zu<br>☐ Trifft teils zu, teils nicht<br>☐ Trifft weitgehend nicht zu<br>☐ Trifft überhaupt nicht zu<br>☐ Kann ich nicht beurteilen
**Ich fürchte finanzielle Einbußen durch vorübergehende Schließung meiner Praxis im Falle einer Infektion durch SARS-CoV-2.**	☐ Ja<br>☐ Nein

© Universitätsklinikum Heidelberg 2020

Part 3: Knowledge and sources of knowledge related to the SARS-CoV-2 pandemic

Wissensquellen/ Wissensstand	
**Bezüglich SARS-CoV-2 bin ich derzeit auf dem aktuellen Wissensstand.**	☐ Trifft ganz zu<br>☐ Trifft weitgehend zu<br>☐ Trifft teils zu, teils nicht<br>☐ Trifft weitgehend nicht zu<br>☐ Trifft überhaupt nicht zu<br>☐ Kann ich nicht beurteilen
**Den Aufwand, eigene Wissenslücken bzgl. SARS-CoV-2 zu schließen, habe ich zu Beginn der SARS-CoV-2-Pandemie als hoch eingeschätzt.**	☐ Trifft ganz zu<br>☐ Trifft weitgehend zu<br>☐ Trifft teils zu, teils nicht<br>☐ Trifft weitgehend nicht zu<br>☐ Trifft überhaupt nicht zu<br>☐ Kann ich nicht beurteilen
**Den Aufwand, eigene Wissenslücken bzgl. SARS-CoV-2 zu schließen, schätze ich derzeit als hoch ein.**	☐ Trifft ganz zu<br>☐ Trifft weitgehend zu<br>☐ Trifft teils zu, teils nicht<br>☐ Trifft weitgehend nicht zu<br>☐ Trifft überhaupt nicht zu<br>☐ Kann ich nicht beurteilen
**Informationen, die ich hierfür nutze, sind:**	☐ Kassenärztliche Vereinigung<br>☐ Robert-Koch-Institut (RKI)<br>☐ Gesundheitsamt<br>☐ Deutsche Gesellschaft für Allgemeinmedizin (DEGAM)<br>☐ Hausärzteverband (HÄV)<br>☐ Kolleg*innen<br>☐ Ärztekammer<br>☐ Deutsches Ärzteblatt<br>☐ Deximed<br>☐ Podcasts<br>○ NDR Prof. Drosten<br>○ MDR Prof. Kekulé<br>○ Ärztezeitung Prof. Scherer<br>○ Sonstige<br>☐ Medizinische Journals<br>☐ Sonstige: ________________________________<br>☐

Part 4: Self-efficacy regarding anamnestic and diagnostic procedures related to SARS-CoV-2

Anamnese und Diagnostik	
**<u>Zu Beginn der SARS-CoV-2-Pandemie</u> habe ich mich in der Anamnese und Diagnostik bei PAT_COV sicher gefühlt.**	☐ Trifft ganz zu<br>☐ Trifft weitgehend zu<br>☐ Trifft teils zu, teils nicht<br>☐ Trifft weitgehend nicht zu<br>☐ Trifft überhaupt nicht zu<br>☐ Kann ich nicht beurteilen
**<u>In der Osterzeit 2020</u> habe ich mich in der Anamnese und Diagnostik bei PAT_COV sicher gefühlt.**	☐ Trifft ganz zu<br>☐ Trifft weitgehend zu<br>☐ Trifft teils zu, teils nicht<br>☐ Trifft weitgehend nicht zu<br>☐ Trifft überhaupt nicht zu<br>☐ Kann ich nicht beurteilen
**<u>Derzeit</u> fühle ich mich in der Anamnese und Diagnostik bei PAT_COV sicher.**	☐ Trifft ganz zu<br>☐ Trifft weitgehend zu<br>☐ Trifft teils zu, teils nicht<br>☐ Trifft weitgehend nicht zu<br>☐ Trifft überhaupt nicht zu<br>☐ Kann ich nicht beurteilen
**<u>Zu Beginn der SARS-CoV-2-Pandemie</u> habe ich mich in der Entscheidung des Procedere bei PAT_COV sicher gefühlt.**<br><br>*Procedere: Therapie, ambulante oder stationäre Weiterbehandlung, nächste Wiedervorstellung*	☐ Trifft ganz zu<br>☐ Trifft weitgehend zu<br>☐ Trifft teils zu, teils nicht<br>☐ Trifft weitgehend nicht zu<br>☐ Trifft überhaupt nicht zu<br>☐ Kann ich nicht beurteilen
**<u>In der Osterzeit 2020</u> fühlte ich mich in der Entscheidung des Procedere bei PAT_COV sicher.**<br><br>*Procedere: Therapie, ambulante oder stationäre Weiterbehandlung, nächste Wiedervorstellung*	☐ Trifft ganz zu<br>☐ Trifft weitgehend zu<br>☐ Trifft teils zu, teils nicht<br>☐ Trifft weitgehend nicht zu<br>☐ Trifft überhaupt nicht zu<br>☐ Kann ich nicht beurteilen
**<u>Derzeit</u> fühle ich mich in der Entscheidung des Procedere bei PAT_COV sicher.**<br><br>*Procedere: Therapie, ambulante oder stationäre Weiterbehandlung, nächste Wiedervorstellung*	☐ Trifft ganz zu<br>☐ Trifft weitgehend zu<br>☐ Trifft teils zu, teils nicht<br>☐ Trifft weitgehend nicht zu<br>☐ Trifft überhaupt nicht zu<br>☐ Kann ich nicht beurteilen

© Universitätsklinikum Heidelberg 2020

Part 5: Prevention measures to contain the spread of SARS-CoV-2

**Maßnahmen zur Eindämmung der SARS-CoV-2 Pandemie**	
**Die Umsetzung der Hygienevorschriften bei Kontakt mit PAT_COV ist in unserer Einrichtung gut umsetzbar.**	☐ Trifft ganz zu<br>☐ Trifft weitgehend zu<br>☐ Trifft teils zu, teils nicht<br>☐ Trifft weitgehend nicht zu<br>☐ Trifft überhaupt nicht zu<br>☐ Kann ich nicht beurteilen
**Die Distanzierung von PAT_COV gelingt in unserer Hausarztpraxis <u>räumlich.</u>**	☐ Trifft ganz zu<br>☐ Trifft weitgehend zu<br>☐ Trifft teils zu, teils nicht<br>☐ Trifft weitgehend nicht zu<br>☐ Trifft überhaupt nicht zu<br>☐ Kann ich nicht beurteilen
**Wie führen Sie die räumliche Trennung genau durch?**<br><br>*(Mehrfachnennung möglich)*	☐ Unterschiedliche Wartezimmer<br>☐ Unterschiedliche Behandlungsräume<br>☐ Wartezeit im eigenen Auto<br>☐ Wartezeit vor der Einrichtung<br>☐ Wegemarkierungen<br>☐ Sonstige: ______________________________<br>Anmerkungen:
**Die Distanzierung von PAT_COV gelingt in unserer Hausarztpraxis <u>zeitlich.</u>**	☐ Trifft ganz zu<br>☐ Trifft weitgehend zu<br>☐ Trifft teils zu, teils nicht<br>☐ Trifft weitgehend nicht zu<br>☐ Trifft überhaupt nicht zu<br>☐ Kann ich nicht beurteilen
**Wie führen Sie die zeitliche Trennung genau durch?**<br><br>*(Mehrfachnennung möglich)*	☐ Bestimmte Sprechstundenzeiten für PAT_COV<br>☐ Weniger Patient*innen ingesamt<br>☐ Bestimmte Wochentage ausschließlich für PAT_COV<br>☐ Sonstige: ______________________________<br>Anmerkungen:

© Universitätsklinikum Heidelberg 2020

Part 6: Treatment capacity per SARS-CoV-2 contact point and primary care practices during the first wave of the SARS-CoV-2 pandemic in Baden-Wuerttemberg, Germany

**Versorgung von Patient*innen mit Verdacht auf oder gesicherte COVID-19-Erkrankung (PAT_COV)**	
**Wie viele PAT_COV pro Tag haben Sie seit März 2020 behandelt?**<br>*(es zählt auch eine alleinige Abstrichentnahme als Behandlung)*	
**Durchschnittlich Tag**	
**Gesamt seit Beginn der SARS-Cov-2-Pandemie 2020 im März 2020**	

© Universitätsklinikum Heidelberg 2020

Questionnaire: Corona-Subspecialised Primary Care Practices

Part 1: Demographic and Practice Characteristics

**Bitte geben Sie das heutige Datum an.**<br>*z.B. 12.03.2020*	
**Soziodemographie**	
**Welcher Altersgruppe gehören Sie an?**	☐ < 30 Jahre<br>☐ 30 bis 40 Jahre<br>☐ 41 bis 50 Jahre<br>☐ 51 bis 60 Jahre<br>☐ 61 bis 70 Jahre<br>☐ > 70 Jahre
**Welchem Geschlecht fühlen Sie sich zugehörig?**	☐ Weiblich<br>☐ Männlich<br>☐ Divers<br>☐ Andere: ______________________________

**Struktur/Organisation**	
**In welcher Corona-Anlaufstelle sind Sie tätig?**<br>*Bitte berücksichtigen Sie in dieser Studie nur Ihre Tätigkeit in nebenstehender Einrichtung. Weitere Tätigkeitsfelder (z.B. Hausarztpraxen) werden gesondert befragt.*	☐ Zentrale Fieberambulanz<br>☐ Corona-Schwerpunktpraxis<br>☐ Abstrichstelle
**Wie viele Einwohner*innen hat der Ort, in dem sich Ihre Corona-Anlaufstelle befindet?**	☐ < 5000 Einwohner*innen<br>☐ 5000 - < 20.000 Einwohner*innen<br>☐ 20.000 - < 100.000 Einwohner*innen<br>☐ 100.000 und mehr Einwohner*innen<br>☐ Kann ich nicht beurteilen
**Wie groß ist der Radius des hauptsächlichen Einzugsgebiets Ihrer Corona-Anlaufstelle?**	☐ < 15 km<br>☐ 15 - < 30 km<br>☐ 30 - < 50 km<br>☐ 50 km oder mehr<br>☐ Kann ich nicht beurteilen
**Wo liegt Ihre Corona-Anlaufstelle?**	☐ Stadtzentrum<br>☐ Stadt-Umkreis (20km)<br>☐ Ländliches Gebiet (Stadt > 20km)
**Wann wurde Ihre Corona-Anlaufstelle in Betrieb genommen?**<br>*(um Ihre Anonymität zu wahren bitten wir Sie nur den Monat der Eröffnung einzutragen)*	

© Universitätsklinikum Heidelberg 2020

**Welche berufliche Qualifikation haben Sie?**<br><br>*(Mehrfachnennung möglich)*	☐ Ärztin/ Arzt ohne Gebietsbezeichnung<br>☐ Fachärztin/-arzt für Allgemeinmedizin<br>☐ *mit aktuell hausärztlicher Tätigkeit*<br>☐ *ohne aktuell hausärztliche Tätigkeit*<br>☐ Fachärztin/-arzt für Innere Medizin<br>☐ *mit aktuell hausärztlicher Tätigkeit*<br>☐ *ohne aktuell hausärztliche Tätigkeit*<br>☐ Fachärztin/ Facharzt für: ____________________<br>☐ Andere: ____________________

Part 2: Fear for a potential infection with SARS-CoV-2 or financial loss due to practice closure

**Einstellung/ Haltung**	
**Ich habe Angst, mich bei meiner Tätigkeit mit SARS-CoV-2 anzustecken, weil ich <u>selbst erkranken</u> könnte.**	☐ Trifft ganz zu<br>☐ Trifft weitgehend zu<br>☐ Trifft teils zu, teils nicht<br>☐ Trifft weitgehend nicht zu<br>☐ Trifft überhaupt nicht zu<br>☐ Kann ich nicht beurteilen
**Ich gehöre einer Risikogruppe für einen schweren Verlauf bei Infektion oder Erkrankung durch SARS-CoV-2 an**	☐ Ja<br>☐ Nein
**Ich habe Angst, mich bei meiner Tätigkeit mit SARS-CoV-2 anzustecken, weil ich andere im <u>privaten</u> Umfeld anstecken könnte.**	☐ Trifft ganz zu<br>☐ Trifft weitgehend zu<br>☐ Trifft teils zu, teils nicht<br>☐ Trifft weitgehend nicht zu<br>☐ Trifft überhaupt nicht zu<br>☐ Kann ich nicht beurteilen
**Ich habe Angst, mich bei meiner Tätigkeit mit SARS-CoV-2 anzustecken, weil ich andere im <u>beruflichen</u> Umfeld (Patient*innen, Mitarbeitende) anstecken könnte.**	☐ Trifft ganz zu<br>☐ Trifft weitgehend zu<br>☐ Trifft teils zu, teils nicht<br>☐ Trifft weitgehend nicht zu<br>☐ Trifft überhaupt nicht zu<br>☐ Kann ich nicht beurteilen
**Ich fürchte finanzielle Einbußen durch vorübergehende Schließung meiner Praxis im Falle einer Infektion durch SARS-CoV-2.**	☐ Ja<br>☐ Nein

© Universitätsklinikum Heidelberg 2020

Part 3: Knowledge and sources of knowledge related to the SARS-CoV-2 pandemic

Wissensquellen/ Wissensstand	
**Bezüglich SARS-CoV-2 bin ich derzeit auf dem aktuellen Wissensstand.**	☐ Trifft ganz zu<br>☐ Trifft weitgehend zu<br>☐ Trifft teils zu, teils nicht<br>☐ Trifft weitgehend nicht zu<br>☐ Trifft überhaupt nicht zu<br>☐ Kann ich nicht beurteilen
**Den Aufwand, eigene Wissenslücken bzgl. SARS-CoV-2 zu schließen, habe ich zu Beginn der SARS-CoV-2-Pandemie als hoch eingeschätzt.**	☐ Trifft ganz zu<br>☐ Trifft weitgehend zu<br>☐ Trifft teils zu, teils nicht<br>☐ Trifft weitgehend nicht zu<br>☐ Trifft überhaupt nicht zu<br>☐ Kann ich nicht beurteilen
**Den Aufwand, eigene Wissenslücken bzgl. SARS-CoV-2 zu schließen, schätze ich derzeit als hoch ein.**	☐ Trifft ganz zu<br>☐ Trifft weitgehend zu<br>☐ Trifft teils zu, teils nicht<br>☐ Trifft weitgehend nicht zu<br>☐ Trifft überhaupt nicht zu<br>☐ Kann ich nicht beurteilen
**Informationen, die ich hierfür nutze, sind:**	☐ Kassenärztliche Vereinigung<br>☐ Robert-Koch-Institut (RKI)<br>☐ Gesundheitsamt<br>☐ Deutsche Gesellschaft für Allgemeinmedizin (DEGAM)<br>☐ Hausärzteverband (HÄV)<br>☐ Kolleg*innen<br>☐ Ärztekammer<br>☐ Deutsches Ärzteblatt<br>☐ Deximed<br>☐ Podcasts<br>o NDR Prof. Drosten<br>o MDR Prof. Kekulé<br>o Ärztezeitung Prof. Scherer<br>o Sonstige<br>☐ Medizinische Journals<br>☐ Sonstige: ______________________

Part 4: Self-efficacy regarding anamnestic and diagnostic procedures related to SARS-CoV-2

Anamnese und Diagnostik	
**Zu Beginn meiner Tätigkeit in der Corona-Anlaufstelle habe ich mich in der Anamnese und Diagnostik bei PAT_COV sicher gefühlt.**	☐ Trifft ganz zu<br>☐ Trifft weitgehend zu<br>☐ Trifft teils zu, teils nicht<br>☐ Trifft weitgehend nicht zu<br>☐ Trifft überhaupt nicht zu<br>☐ Kann ich nicht beurteilen
**Nach 4 Wochen meiner Tätigkeit in der Corona-Anlaufstelle habe ich mich in der Anamnese und Diagnostik bei PAT_COV sicher gefühlt.**	☐ Trifft ganz zu<br>☐ Trifft weitgehend zu<br>☐ Trifft teils zu, teils nicht<br>☐ Trifft weitgehend nicht zu<br>☐ Trifft überhaupt nicht zu<br>☐ Kann ich nicht beurteilen
**Derzeit fühle ich in der Anamnese und Diagnostik bei PAT_COV sicher.**	☐ Trifft ganz zu<br>☐ Trifft weitgehend zu<br>☐ Trifft teils zu, teils nicht<br>☐ Trifft weitgehend nicht zu<br>☐ Trifft überhaupt nicht zu<br>☐ Kann ich nicht beurteilen
**Zu Beginn meiner Tätigkeit in der Corona-Anlaufstelle fühlte ich mich in der Entscheidung des Procedere bei PAT_COV sicher.**<br><br>*Procedere: Therapie, ambulante oder stationäre Weiterbehandlung, nächste Wiedervorstellung*	☐ Trifft ganz zu<br>☐ Trifft weitgehend zu<br>☐ Trifft teils zu, teils nicht<br>☐ Trifft weitgehend nicht zu<br>☐ Trifft überhaupt nicht zu<br>☐ Kann ich nicht beurteilen
**Nach 4 Wochen meiner Tätigkeit in der Corona-Anlaufstelle fühlte ich mich in der Entscheidung des Procedere bei PAT_COV sicher.**<br><br>*Procedere: Therapie, ambulante oder stationäre Weiterbehandlung, nächste Wiedervorstellung*	☐ Trifft ganz zu<br>☐ Trifft weitgehend zu<br>☐ Trifft teils zu, teils nicht<br>☐ Trifft weitgehend nicht zu<br>☐ Trifft überhaupt nicht zu<br>☐ Kann ich nicht beurteilen
**Derzeit fühle ich mich in der Entscheidung des Procedere bei PAT_COV sicher.**<br><br>*Procedere: Therapie, ambulante oder stationäre Weiterbehandlung, nächste Wiedervorstellung*	☐ Trifft ganz zu<br>☐ Trifft weitgehend zu<br>☐ Trifft teils zu, teils nicht<br>☐ Trifft weitgehend nicht zu<br>☐ Trifft überhaupt nicht zu<br>☐ Kann ich nicht beurteilen

© Universitätsklinikum Heidelberg 2020

Part 5: Prevention measures to contain the spread of SARS-CoV-2

Maßnahmen zur Eindämmung der SARS-CoV-2 Pandemie	
**Die Umsetzung der Hygienevorschriften bei Kontakt mit PAT_COV ist in unserer Einrichtung gut umsetzbar.**	☐ Trifft ganz zu<br>☐ Trifft weitgehend zu<br>☐ Trifft teils zu, teils nicht<br>☐ Trifft weitgehend nicht zu<br>☐ Trifft überhaupt nicht zu<br>☐ Kann ich nicht beurteilen
**Die Distanzierung von PAT_COV gelingt in unserer Corona-Anlaufstelle <u>räumlich.</u>**	☐ Trifft zu<br>☐ Trifft eher zu<br>☐ Teils-Teils<br>☐ Trifft eher nicht zu<br>☐ Trifft nicht zu<br>☐ Kann ich nicht beurteilen
**Wie führen Sie die räumliche Trennung genau durch?**<br>*(Mehrfachnennung möglich)*	☐ Unterschiedliche Wartezimmer<br>☐ Wartezeit im eigenen Auto<br>☐ Wartezeit vor der Einrichtung<br>☐ Unterschiedliche Behandlungsräume<br>☐ Wegemarkierungen<br>☐ Sonstige: ______________________________
**Die Distanzierung von PAT_COV gelingt in unserer Corona-Anlaufstelle <u>zeitlich.</u>**	☐ Trifft zu<br>☐ Trifft eher zu<br>☐ Teils-Teils<br>☐ Trifft eher nicht zu<br>☐ Trifft nicht zu<br>☐ Kann ich nicht beurteilen
**Wie führen Sie die zeitliche Trennung genau durch?**<br>*(Mehrfachnennung möglich)*	☐ Terminvergabe<br>☐ Sonstige: ______________________________<br>Anmerkungen:

© Universitätsklinikum Heidelberg 2020

Part 6: Treatment capacity per SARS-CoV-2 contact point and primary care practices during the first wave of the SARS-CoV-2 pandemic in Baden-Wuerttemberg, Germany

<table>
<tr><th colspan="2">Patientenkontakte/Kapazität</th></tr>
<tr><td colspan="2">Wie viele Patient*innen mit Verdacht auf oder gesicherter COVID-19-Erkrankung (PAT_COV) <u>haben Sie seit Eröffnung</u> behandelt?

(es zählt auch eine alleinige Abstrich-Entnahme als Behandlung)</td></tr>
<tr><td>Durchschnittlich Tag</td><td></td></tr>
<tr><td>Gesamt seit Beginn der SARS-Cov-2-Pandemie 2020 im März 2020</td><td></td></tr>
<tr><td>Wie viele PAT_COV <u>könnten Sie</u> pro Tag in Ihrer Corona-Anlaufstelle maximal behandeln?

(bitte tragen Sie die jeweilige Anzahl auf den Linien ein)</td><td>_________ sofort
_________, wenn ich personell aufstocke
_________, wenn ich weitere Maßnahmen ergreife</td></tr>
</table>

© Universitätsklinikum Heidelberg 2020
